# Neural progenitor cell transplantation results in structural and functional recovery in a rat model of cerebral palsy

**DOI:** 10.22038/ijbms.2025.85616.18508

**Published:** 2025

**Authors:** Maryam Tavakoli Lakeh, Seyed Massood Nabavi, Fatemeh Rouhollah, Koorosh Shahpasand, Sahar Kiani

**Affiliations:** 1 Department of Cellular and Molecular Biology, Faculty of Advanced Sciences and Technology, Tehran Medical Sciences, Islamic Azad University, Tehran, Iran; 2 Department of Regenerative Medicine, Cell Science Research Center, Royan Institute for Stem Cell Biology and Technology, ACECR, Tehran, Iran; 3 Department of Laboratory Medicine and Pathology, University of Minnesota Medical School, Minneapolis, Minnesota, United States; 4 Department of Stem Cell and Developmental Biology, Cell Science Research Center, ROYAN Institute for Stem Cell Biology and Technology, ACECR, Tehran, Iran; 5 Department of Brain and Cognitive Sciences, Cell Science Research Center, ROYAN Institute for Stem Cell Biology and Technology, ACECR, Tehran, Iran

**Keywords:** Cell transplantation, Cerebral palsy, Hypoxia, Rats, Stem cells

## Abstract

**Objective(s):**

Cerebral palsy (CP) is the most prevalent pediatric neurodevelopmental disorder. Stem cell therapy is a promising way to treat brain disorders, including CP. This study sought to establish a model using pregnant rats to induce CP similarly to that observed in humans. This approach aims to enhance our understanding of the mechanisms underlying CP and explores the potential for healing brain injuries through the transplantation of neural progenitor cells (NPCs).

**Materials and Methods:**

In this experimental study, stress conditions were induced to create a CP model in neonatal rats. Initially, the uterine vein was blocked in pregnant rats to induce hypoxic conditions. Consequently, histological analyses are performed to assess the extent of brain damage in rats.

**Results:**

The findings indicated that the CP group exhibited notable pathological alterations, as shown by histochemical analysis, which revealed lesions in the cortical brain tissues of neonatal rats. After confirming our CP model, NPCs were transplanted into the motor cortex of CP neonates (PND7) by microinjection. After two days, the neonates were sacrificed, and the brain tissue was pathologically analyzed. Our study shows that transplantation of neural progenitor cells decreases inflammation and regulation of astrogenesis.

**Conclusion:**

The induction of hypoxia-ischemia (HI) in the uterus appears to be a reliable animal model for studying CP mechanisms. Additionally, our research demonstrates that the transplantation of NPCs is a promising therapeutic approach for treating CP. This advancement will enhance our comprehension and aid in the refinement of cellular therapeutic strategies.

## Introduction

Cerebral palsy (CP) is the leading cause of motor impairments in infants and children (1). It is induced by an injury to the developing brain that occurs before, during, or after birth in about 1%–3% of the population by the age of 2 years (2, 3). According to a recent report, CP involves about 2 to 4 cases per 1000 live births worldwide. Also, the prevalence of this disorder has been reported to be approximately 2 cases per 1000 live births in Iran (4). Cerebral palsy is commonly linked to a range of deficits, which may include sensory, perceptual, and cognitive abnormalities, mental retardation, language and communication disorders, behavioral disturbances, seizures, and subsequent musculoskeletal problems (5, 6). 

CP describes a group of permanent disorders of development, movement, and posture that cause activity limitation (7). It is linked to a variety of mechanisms and neural pathways that influence the type and severity of motor disability (8). Hypoxia-ischemia (HI) is recognized as a leading factor contributing to CP and has played a crucial role in the creation of animal models designed to replicate CP (9, 10). Inadequate oxygenation in children with CP leads to delayed brain development, resulting in secondary cellular vulnerability to hypoxic-ischemic injury (11, 12). Neonatal HI, caused by oxygenation and blood flow disruption during prenatal and postnatal periods, results in nitrative stress, inflammatory responses, and white matter (WM) loss, affecting oligodendroglia (13). According to Zaghloul *et al*. (14), WM in mice shows greater sensitivity to HI injury than gray matter. Their histological analysis revealed WM atrophy, microglial activation, and neuronal death (15). Over the past few decades, research using HI rat models has uncovered remarkably analogous mechanisms of brain injury and damage progression in rodents and human neonates affected by CP. The apoptotic-necrotic pattern of damage in both white and gray matter, alongside neuromotor abnormalities, results from hypoxia and can be reliably replicated using precisely calibrated HI models (16).

Conversely, the use of HI rat models allows for the reproduction of brain injury during a crucial developmental phase similar to that experienced by humans. This critical phase aligns between 24 and 32 weeks of gestation in humans and postnatal day (PND) 2-5 in rodent models. These rodent models have played a crucial role in the investigation of CP because of their ability to exhibit brain injury, HI conditions, inflammation, and consequential motor and cognitive impairments (17). Significant progress has been made in developing HI rodent models designed to more accurately replicate the pathophysiological mechanisms that contribute to brain damage and the subsequent effects seen in patients with CP (17, 18). The CP model in Race-Vannucci mice, a well-established approach, has been widely applied in numerous animal model studies concerning neonatal HI. In this methodology, the unilateral occlusion of the common carotid artery in 7-day-old rats is followed by exposure to controlled hypoxic conditions containing 8% oxygen (19).

Also, researchers established unilateral ischemic reperfusion in pregnant mice and examined inflammatory markers in the embryos at the molecular level. However, our method analyzed behavioral responses and histology in offspring on various postnatal days. Additionally, in another study, investigators induced hypoperfusion in rat embryos using the micro coil stenosis method, resulting in decreased blood flow in both the ovary and placenta. This form of occlusion diverged from our temporary method (19, 20). Our approach increases embryo viability risk, facilitating a straightforward and precise examination of outcomes. Various factors, including the duration and nature of vessel occlusion, the species involved (mice or rats), and the types of instruments used, affect the results.

According to recent findings, a hypoxic state in the last months of pregnancy is the most common reason for CP in humans (21). By considering the most important factors in CP, our experimental study was performed with an emphasis on CP modeling in humans by inducing hypoxia in pregnant rats in the last week of pregnancy to be a new strategy to help understand more about the mechanisms of CP. 

On the other hand, various clinical treatments have been used for ischemic brain injury, such as hyperbaric oxygen, medications (22), rehabilitation training (23), and therapeutic hypothermia (24). However, most of these therapeutic methods are supportive measures that have no therapeutic effect for hypoxic-ischemic brain injury, and none can reverse the consequences (25). With the emergence of stem cell biology, stem cell therapy has been a strategy to restore function in ischemic brain injury (26, 27). However, most of these therapeutic methods are supportive measures that have no effective treatments for hypoxic-ischemic brain injury (28).

The capacity of stem cells for differentiation and self-renewal enables them to generate different functional cells and migrate into the damaged areas of the brain (29). Stem cell-based therapies have emerged as an encouraging approach for treating numerous neurological disorders with the potential for stem cell regeneration to restore damaged neural tissue (30). The stem cells’ ability to differentiate and self-renew allows them to form various functional cells and migrate into the injured brain regions (31, 32). Neural Progenitor cells (NPCs) are somatic stem cells that first generate the radial glial progenitor cells, which create the neurons, astrocytes, and oligodendrocytes in the Central Nervous System (CNS) (33). Moreover, these transplanted neural stem cells can secrete neurotrophic factors that promote the survival of residual endogenous cells and endogenous neurogenesis (34). Neural stem cells could modulate the inflammatory milieu of the ischemic brain by releasing anti-inflammatory cytokines. These characteristics position them as a potentially effective therapy for hypoxic-ischemic disorders in the future (35).

## Materials and Methods

### Preparing the CP model

This experimental study has been approved by the Research Ethics Committee of Islamic Azad University, Tehran Medical Branch, and the Royan Institute of Iran (IR.ACECR.ROYAN.REC.1399.085). Ethical guidelines for research on laboratory animals were considered in all experimental processes. A total of 42-week-aged pregnant Wistar rats (E17; weight=350-400 g) were allocated into the sham group (n=2) and the model group (n=2). The rats had free access to food and water in a room with a 12-hour day/night cycle, a temperature of 23 °C to 25 °C, humidity between 45% and 60%, and regular disinfection and ventilation. 

**Figure 1:**
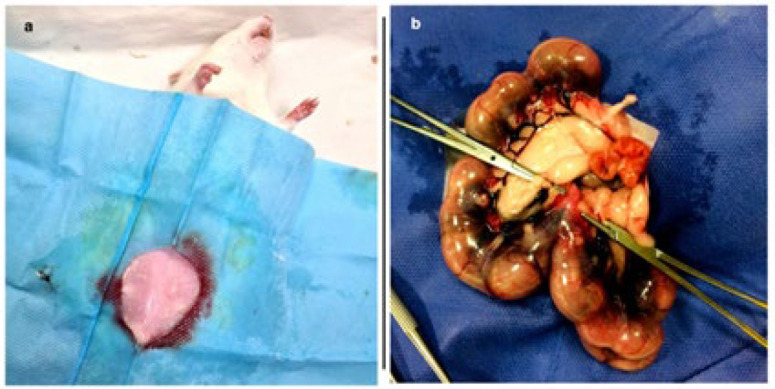
The rat was placed in the surgical area, and an incision was made in the abdomen according to conventional disinfection methods

At E17, pregnant rats were subjected to either sham surgery or HI surgery. Each rat was anesthetized by the IP injection of ketamine (100 mg/kg) and medetomidine (0.5 mg/kg). Subsequently, as illustrated in Figure 1, the rats were positioned at the surgical site. Following conventional disinfection, an incision was made in the rat’s abdomen, and the uterine artery was exposed and clamped for ten minutes by patch. In the sham group, only the laparotomy was performed without uterine manipulation. The total surgical procedure took about 15 min. Following the surgical procedure, the rats recovered and returned to their cage, and four days after surgery, the offspring were delivered. On postnatal day (PND) 1, 3, 5, 7, and 14, the brain tissues of experimental neonatal rats were sectioned for histological evaluations (n=3).

**Figure 2:**
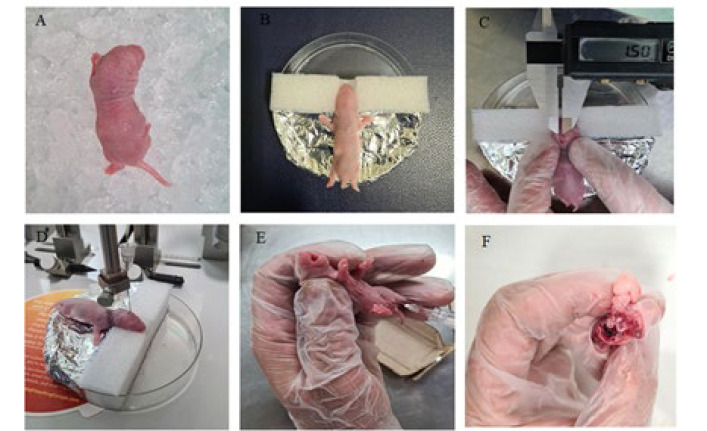
Newborn rats were anesthetized with reduced body temperature by ice (A), The rats' heads were fixed in the designed location (B), The coordinates were determined and marked with a caliper (C), The NPCs were transplanted using the microinjection of the Stereotaxic device (D), Two days after cell injection, the rats were anesthetized (E), The rat brains were removed and placed in formalin (F). NPCs: Neural progenitor cells.

Following the confirmation of CP modeling via histological analysis conducted by a pathobiologist, we proceeded to replicate the previously mentioned surgical procedure on an additional pregnant rat to advance our research. After surgery, on postnatal day 7, rat neonates were allocated into two groups. The appearance of the cellular characteristics of CP in the PND7 group was more noticeable than in other groups. The first CP group (n=5) received NPC transplantation; the second group (n=5) served as a control and did not receive NPC transplantation. The study’s design is illustrated in Figure 2.

### Culture of neural progenitor cell (NPC) in 2D monolayer culture

**Figure 3:**
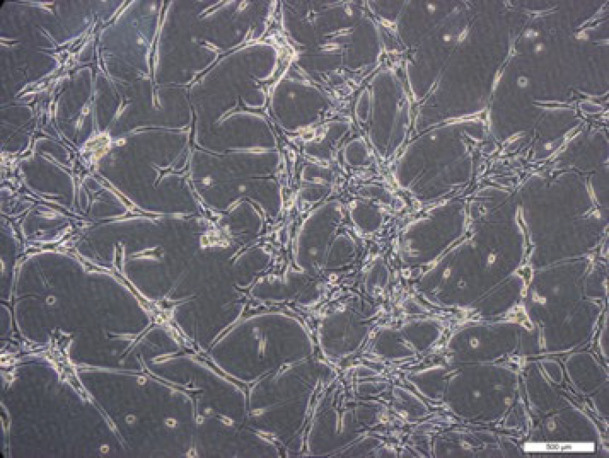
Expanded neural progenitor cells (NPC) on Laminin/Poly-L-ornithine coated dishes in NPC medium

According to published protocols, neural progenitor cells (NPC) were isolated from the embryonic neocortex. Neural stem cell isolation and *in vitro* analyses were done by Dr. Homayouni Moghadam’s laboratory at Royan Research Institute of Isfahan (36). All necessary analyses were performed to confirm the existence of neural progenitor cells (NPCs) in his laboratory. We expanded neural stem cells obtained from the Royan Research Institute of Isfahan (Figure 3).

### Histochemical analysis

The rats in the CP and sham groups were anesthetized and perfused transcardially with 0.1 molar Phosphate Buffered saline (PBS) on PNDs 1, 3, 5, 7, and 14. Their brains were detached and fixed with 10% formalin (H&E and Nissl staining) and 4% paraformaldehyde (IHC staining) for 72 hr at room temperature. The brains were embedded in paraffin and were sliced into coronal 5 μm blocks collected on albumin-subbed slides. Afterward, the slides were dried in an incubator overnight.

### Hematoxylin and eosin staining

 Hematoxylin and Eosin (H&E) is an appropriate staining for the nucleus and cytoplasm with contrasting colors to distinguish cellular components. According to the standard published protocols, tissue sections were stained by H&E for anatomical pathology diagnosis (37, 38). Briefly, the tissues were soaked in xylene for paraffin removal. Subsequently, a series of ethanol gradients at concentrations of 100%, 96%, and 70% were employed to dehydrate the brains. The tissues were stained with hematoxylin solution and then rinsed in tap water. The tissue was soaked in saturated lithium carbonate solution in the bluing step, and the acid alcohol was then rinsed with tap water. Finally, staining was accomplished with an eosin solution. For the H&E staining assessment, composite scores were given as follows: 

0: no pathological changes (without apoptosis, necrosis, and inflammation); 

1: mild pathological changes with no apparent neuronal loss; 

2: moderate pathological changes with possible but mild neuronal loss; 

3: severe injury with widespread pathological changes and neuronal loss.

### Immunohistochemical staining

Brain tissue sections were deparaffinized by heating with xylene and were rehydrated with serial dilutions of ethanol. Two drops (100 μl) of peroxidase-blocking reagent were added and kept at room temperature and in the dark for ten minutes. Sections were incubated overnight at 4 °C with primary antibodies, including rabbit anti-glial fibrillary acidic protein (GFAP) (1:300; #ab7260, Abcam, United Kingdom) as a marker for reactive astrocytosis, mouse anti-neuronal specific nuclear protein (NeuN) (1:200; #ab104224, Abcam, United Kingdom), and mouse anti-rat CD68 (1:100; #MCA341R, Bio-Rad, United States) as a marker for reactive macrophage and monocyte in animals. The sections were incubated with primary antibody, amplifier master, and then with master polymer plus HRP. After each step, the slides were rinsed in Tris-Buffered Saline (TBS) three times for five minutes. Subsequently, the slides were processed using the DAB substrate kit, incubated with substrate/chromogen using Master Diagnostica developing kits (Code No. MAD-000237QK-S), and rinsed three times with distilled water for five minutes. Eventually, the sections were counter-stained with hematoxylin (Sigma-Aldrich, MHS1) for nuclear staining, dehydrated with serial dilutions of ethanol, placed into xylene, and mounted with Entellan (Sigma-Aldrich). Bright illumination field images were obtained using an Olympus BX51 Microscope to quantify positive cells.

### Neural progenitor cell viability

The viable NPC cells were stained with trypan blue and subsequently counted with Neobar lam for verification. A 1 × 106 NPCs/ml confluent layer was stained with CM-Dil dye (Invitrogen; Germany). According to protocol, the stock solution CM-Dil reagent was diluted at 1:20 and 1:400 to obtain 50 and 2.5nM solutions in DMSO. Then, 1 ml of 0.05% trypsin-EDTA was added to the tube and incubated at 37 °C for ten minutes. Subsequently, 2 ml of the prepared CM-Dil was added to NPC cells and incubated for 20 min at 37 °C with moderate stirring. This dye is a red fluorescent dye that can label cells by binding to the cell membrane. Afterward, some cell suspension was placed on the slide and checked under a fluorescent microscope to ensure the correct labeling of the cells. The vials were subsequently placed on ice and moved to the animal laboratory.

### Transplanting cells into the brains of rat neonates

Rat neonates were allocated into two groups, five animals per group (n=5). All newborn rats were anesthetized with reduced body temperature by ice. According to the picture, a place was designed and built to fix the rats’ heads. Due to the small size of the ears and lack of teeth, it was not possible to fix them with the stereotaxic frame (Figure 2-B).

The neonates were then taken to the surgical area, where an incision was made on the top of the head. Injections were made into the left hemisphere of the brain of a seven-day-old rat neonate. NPCs were prepared in a serum-free medium at a density of 5 × 10^4^ live cells/µl and injected with 3 μl cell suspension for NPC transplantation using the microinjection of the Stereotaxic device. The cells were injected at the specified location, the motor cortex, at a specific rate for three minutes (Injection coordinates: Bregma: 0.20 mm and lambda: 20.5 mm. The coordinates were determined and marked with a caliper).

All rat neonates then received a drop of bupivacaine hydrochloride (Marcaine, 5mg/ml, Hospira/Pfizer) applied to the surgical site as an analgesic, and the split was closed with a surgical suture (Sofsilk, Covidien), and the rat neonates recovered under a lamp. The entire surgical procedure took five minutes (Figure 2-C and 1-D). After the rat neonates had recovered and regained consciousness, they were brought back to their mother. Two days after cell injection, the rats were anesthetized using ketamine (100 mg/kg) and xylazine (5 mg/kg) and perfused with 0.9% sodium chloride. Then, their brains were removed and placed in 10% formalin for 72 hr to be fixed (Figures 2-E and 2-F). After fixation, the slides were washed in PBS and then dehydrated in the tissue processor with increasing concentrations of alcohol, embedded in 4% paraformaldehyde, and finally sectioned into serial frontal 5 μM slices using a rotary microtome. The sections were mounted on self-adhesive slides for histopathological tests. The sections underwent histochemical staining with hematoxylin and eosin (H&E) and Nissl stain, as well as immunohistochemical staining with the NeuN antibody, GFAP antibody, and CD68 antibody.

### Statistical analysis

The data were presented as mean ± Standard Deviation (±SD), assuming a normal distribution. The statistical software package SPSS 21.0 was used. N represents the number of animals in each figure, as indicated in the legend of each figure. Statistical significance of the data was assessed using t-test and ANOVA. P-values of 0.05 were considered statistically significant. Data are expressed as mean ± SD; *P<0.05; ** *P*<0.01; *** *P*<0.001; **** *P*<0.0001. All graphs and statistical analyses were created with GraphPad Prism 9.5.1.

## Results

### Histopathological assessments

Tissue structure changes due to brain injury are induced, and H&E staining was used to identify the structure of cells in brain tissue. In continuation, the coronal sections stained with H&E were examined for neuropathology. The results of histopathological examinations are indicated in Tables 1 and 2. According to Table 1, sham groups had normal cellular features in the cerebral cortex, while the degrees of apoptosis and necrosis are noticeable in CP-suspected rats, especially CP4 and CP5.

**Figure 4:**
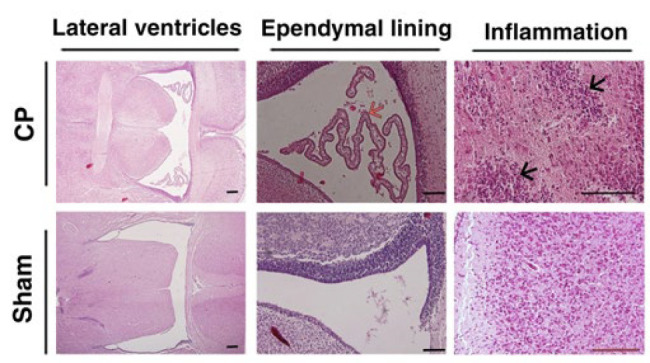
Representative H&E coronal brain sections of lateral ventricle (Left column), the ependymal lining (Middle column), and inflammation (Right column) of the studied groups (CP & Sham groups). Periventricular Leukomalacia (PVL) injury is evident in the CP groups, with markedly enlarged lateral ventricles and a third ventricle compared to sham-treated animals. The black arrows showed inflammation in the section of the CP models. The ependymal lining was shown with a red arrow

Images of brain tissues are also demonstrated in Figure 4. One of the main pathologic features of CP is white matter damage, which can be observed in the observed figures along with structural and cellular abnormal lesions around the ventricle. CP-injured rats showed a variety of ventriculomegaly and neuronal cell death.

**Figure 5:**
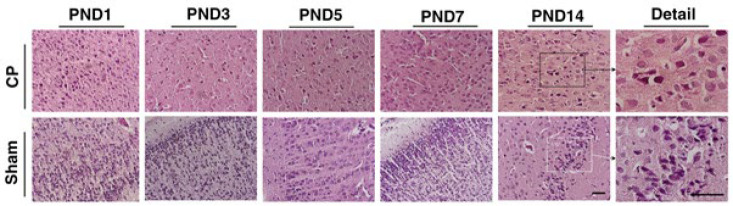
Images of rat cerebral cortex from sham and CP groups stained with hematoxylin and eosin (H&E) at postnatal day (PND) 1, 3, 5, 7, and 14

In Figure 5, the image shows an ischemia pattern in the CP animals (H&E staining). Multifocal periventricular gliosis, periventricular gliosis leukomalacia, and diffused gliosis have been seen in the ventral part of the corpus callosum. Also, the decreased staining for pyramidal cells in CA1 is the ependymal lining, and inflammation was illustrated in Figure 4. 

### Immunohistochemical assessments

**Figure 6:**
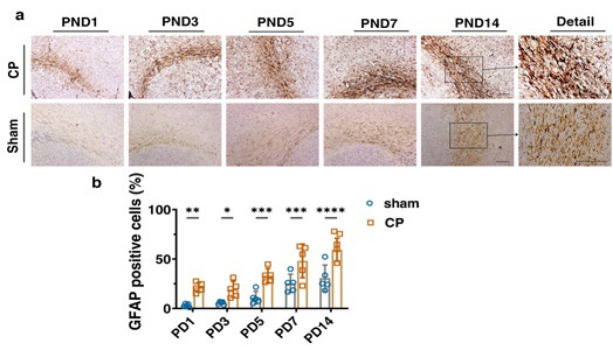
Immunohistochemical staining of rat’s corpus callosum by GFAP marker for sham and CP groups followed by quantification analysis

The GFAP is known as a marker of astrogliosis, and its expression is expanded in CP damage. The immunostaining analysis indicated the significant GFAP accumulation in all five different CP groups in comparison to the sham ones. GFAP increase suggests the activation of astrocytes, which also rises with brain maturity. In the CP group, it was observed that microgliosis augmented with the growth of rats. As PND increases in the CP groups, the severity of microgliosis rises. This difference was more profound in the PND5, PND7, and PND14 groups (Figure 6, Table 3). 

**Figure 7:**
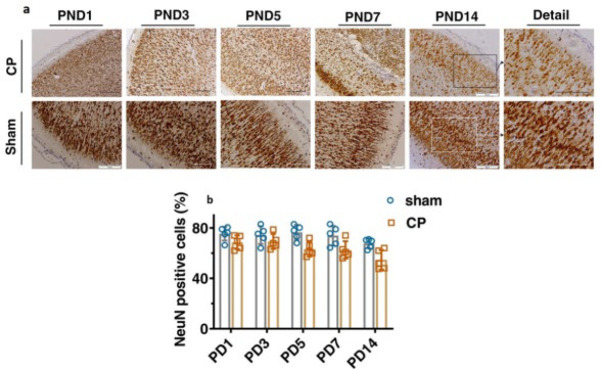
Immunohistochemical staining of rats’ cerebral motor cortices by the NeuN marker for the sham and CP groups

**Figure 8:**
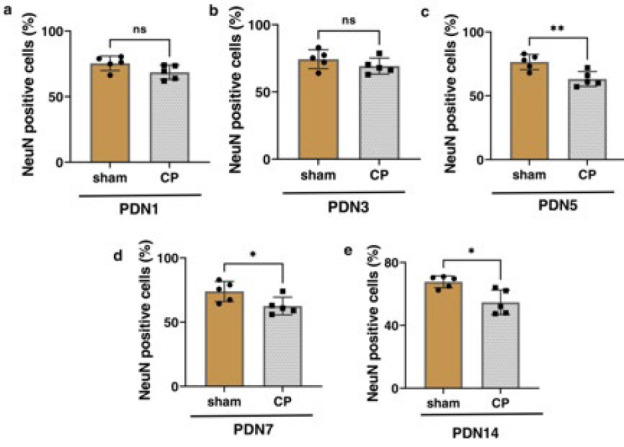
NeuN positive cells percentages in Sham and CP groups at PND1 (a), PND3 (b), PND5 (c), PND7 (d) and PND14 (e)

NeuN antibody was used to identify mature neurons in tissue sections, which was considered a neuronal marker in the motor cortex. Two-way ANOVA showed no significant change in the NeuN marker among different groups (Figure 7, *P*=0.4009). However, the quantification analysis revealed a decrease in the NeuN marker in the cerebral cortices of CP-injured rats in the PND5 (63.22 ± 5.864), PND7 (62.58 ± 6.883), and PND14 (54.69 ± 7.816) groups. Due to the hypoxic conditions in these three CP groups, some neurons were damaged, reducing the number of neurons (Figures 7 and 8 and Table 4).

**Figure 9:**
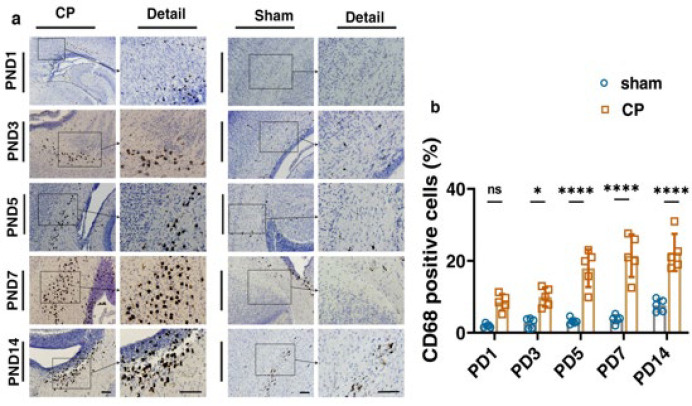
The immunohistochemical staining of rat’s corpus callosum by CD68 marker for the studied samples followed by quantification analysis

CD68 is another antibody used to identify a lysosomal protein commonly found in macrophages and activated microglia. To measure inflammation in the CP-injured samples, we utilized the CD68 marker in this study. The immunostaining showed higher expression of CD68 in the corpus callosum in all five of the CP-injured groups (Figure 9, *F* (4, 16) = 4.534, *P*=0.0122). It has been shown that the resting microglia express CD68 at lower levels. The remarkable amount of CD68 marker implies that activated microglia cells are associated with gliosis and inflammation in the CP groups (Table 5).

Tables 3, 4, 5, and 6 quantitatively state the expression intensity of the mentioned antibodies. The results demonstrated a 1% to 10% increase in immunoreactivity in the sham groups. Conversely, in the CP groups, this value increased to 33% (Tables 3, 4, 5, and 6).

### CM-dil-labeled neuronal progenitor cells

**Figure 10:**
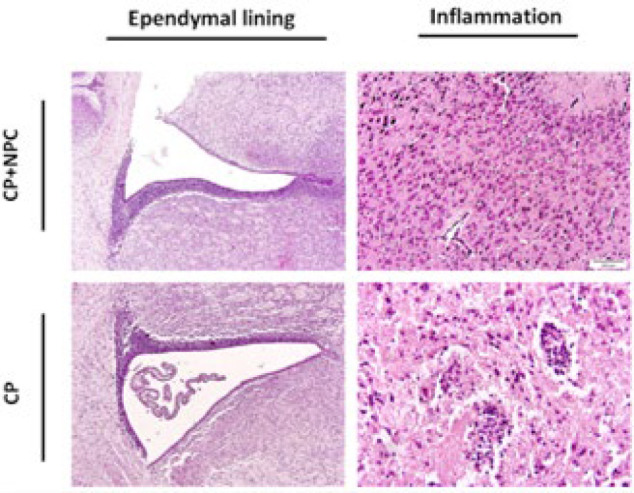
Images of neuronal progenitor cells (NPCs) labeled with CM-Dil at injection area taken by fluorescent microscope different lenses

To verify the attachment and labeling of the cells using CM-Dil dye, a sample of the cell suspension was applied to the slide before its injection into the rat brains, and this was subsequently examined and confirmed using a fluorescence microscope. The staining with CM-Dil fluorescent dye did not affect the viability and morphology of the NPCs. Then, the NPCs labeled with CM-Dil were transferred into the brain and showed a red fluorescent wave. More than 90% of cells were marked with CM-Dil, and the stained cells showed red fluorescence (Figure 10 A, B). 

### Histological analysis after transplantation of cells

**Figure 11:**
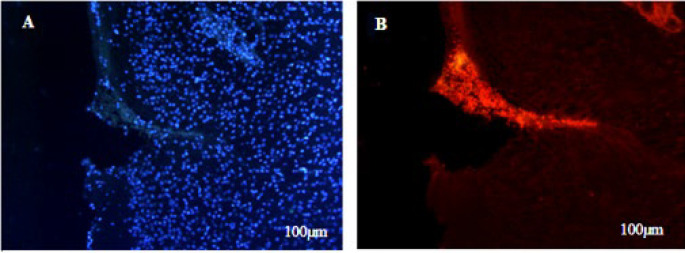
H&E staining of coronal sections of the ependymal lining (Left column) and inflammation (Right column) in the studied samples (CP+NPC and CP groups)

Tissue injuries were analyzed by H&E staining. Coronal sections were stained using H&E and subsequently analyzed through neuropathological examination. The results of histopathological examinations are indicated in Table 7. Ischemic and necrotic cells were less in the group receiving cells with decreased expression than in the CP group (no transplanted NPC) (Figure 11).

### Transplanted NPCs lead to a decrease in Glial fibrillary acidic protein

**Figure 12:**
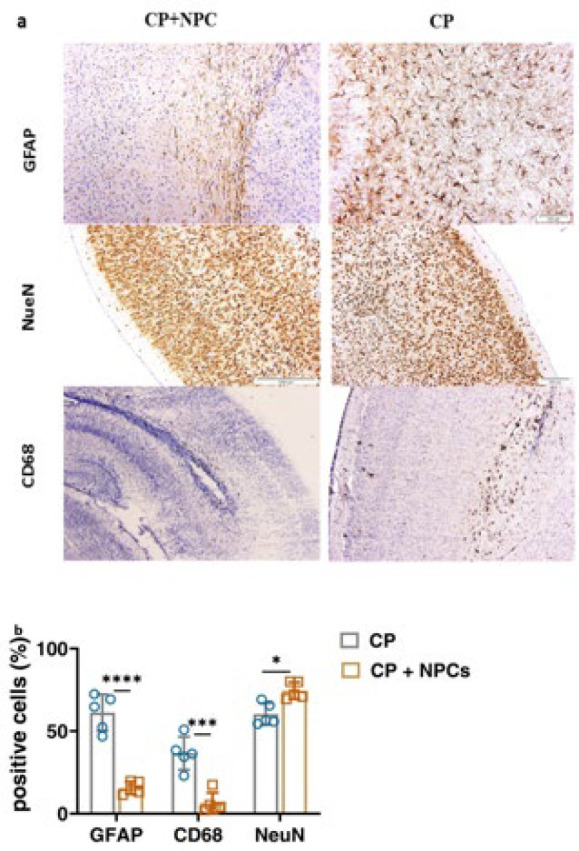
Immunohistochemical staining of rat’s cerebral cortex by GFAP marker, CD68 marker, and NueN marker in the studied samples (CP+NPC and CP groups)

Glial fibrillary acidic protein (GFAP) is a marker for astrogliosis and increases immediately after CNS injury in CP lesions. The increase in GFAP indicates the activation of astrocytes, which also increases with brain maturation (Figure 12). An increase in GFAP means that the severity of gliosis increased; examination of the results of counting and quantitative analysis of this marker showed that the level of GFAP expression was lower in the group that transplanted NPCs than the CP group (Figure 12, GFAP, CP: (61.15 ± 11.04); CP + NPC: (15.59 ± 3.78); *P*<0.0001).

### Differentiated transplanted cells replaced lost neural cells

The NeuN antibody was used as a marker to detect mature neurons in the motor cortex. Two-way analysis of variance showed a significant change in the NeuN in the different groups (Figure 12). The number of neurons in the two groups naturally decreased due to hypoxia. Following cell transplantation, the new cells take the place of the cells that were lost. As a result, the number of neurons in the CP group was lower. Immunostaining showed a significant enhancement in the number of NeuN-positive neurons after transplantation. The group that received compensated cells appears to exhibit a higher expression of the NeuN marker when compared to the group that did not receive new cells. A significant difference in the expression of the NeuN antibody was observed in the two groups, such that the expression of this antibody was higher in the NPC transplantation group. (Figure 12, NeuN, CP: (60.29 ± 6.60); CP + NPC: (74.54 ± 5.10); *P*=0.0456).

### Transplanted NPCs lead to a decrease in inflammation

CD68 is a common marker for identifying a lysosomal protein commonly found in activated macrophages and microglia. CD68 is a marker of inflammation related to the involvement of monocytes/macrophages. In this study, the CD68 marker was used to assess inflammation in different groups. Examination of this staining showed higher expression of CD68 in the CP group, indicating the association of active microglial cells with gliosis and inflammation in CP. However, the expression of CD68 decreased in the group that received the cells, and the injection of new cells led to reduced inflammation in the brain tissue. (Figure 12, CP: (36.59 ± 9.96); CP + NPC: (6.48 ± 6.19); *P* = 0.0006). It seems NPC transplantation successfully decreased inflammation and astrogliosis and enhanced the number of mature neurons in injured treated models (Figure 12, *F* (2, 8) = 44.65, *P*<0.0001).

## Discussion

The current study aimed to create a novel model of CP, which is most similar to the path of induction of CP in humans. White matter erosion is one of the most apparent forms of brain damage (39, 40). These injuries can lead to the nerve’s inflammation. In this study, CP occurred based on oxygen deprivation at a specific point in pregnancy. Earlier research employed this technique to induce CP in certain animals, including rabbits (41). In this research, neonatal rats were chosen due to certain structural similarities between the brains of rats and humans (42). The results of histological analysis revealed that the neuroinflammation evoked in the white matter of the brain caused severe microgliosis. Furthermore, using histopathological examinations, ischemia caused by hypoxia was observed, which led to a decrease in neurons, and the appearance of ischemic cells was observed along with cell necrosis. This destruction demonstrated that the mentioned method can create practical and controlled damage and can make it controllable.

Hypoxia causes augmentation in inflammatory cytokines, which hyperactivate microglia. These processes eventually lead to oligodendrocyte damage and white matter loss in the brain. On the other hand, more than 90% of oligodendrocytes are formed in the white matter of premature infants’ brains, which are more prone to CP. White matter damage, followed by astrogliosis and microgliosis, is due to the impaired maturation of oligodendrocytes (43).

Conversely, a range of clinical approaches has been employed to treat CP; however, these methods have proven ineffective, and there is currently no cure for the condition. Neural progenitor cells can potentially replace damaged neuron cells in neonatal CP. New opportunities exist for developing effective NPC transplantation strategies to provide new neural cells in neurodegenerative disease. Transplanted stem cells can improve tissue regeneration by receiving various signals from the brain microenvironment for migrating to damaged areas, affecting neuronal cell replacement therapy (44). 

In this study, the histopathological examination was performed. The information obtained from the cross-sectioned brain tissues indicated the number of lesions in the hippocampus and ventricles. These results revealed that none of the negative control groups had pathological changes. In contrast, suspected CP groups exhibited histopathological changes (especially in PND 7 and 14). These results can be used to confirm the creation of the CP model. Histological examination also assessed the incidence of inflammation, necrosis, and apoptosis, which measure the degree of white matter destruction of the cerebral cortex. Inflammatory mediators increase in CP due to tissue destruction (45). Brain hypoxia can cause necrotic cells, resulting in increased apoptosis of the neurons.

Moreover, our findings exhibited that the damage rate in the CP groups, especially PND 7 and 14, increased significantly due to inflammation and necrosis (Table 3). Consequently, as illustrated in Figure 3, leukomalacia is present in the periventricular white matter, which, with inflammation in the cortex and periventricular region, can indicate the development of the CP model. In Figure 3, in addition to inflammation, the difference between the two groups is visible in the lateral ventricles. Previous reports have indicated that hypoxia is characterized by an increase in the size of lateral ventricles and the shedding of ependymal lining (18, 21).

After NPC transplantation, the pathological changes were also examined. H&E staining showed fewer ischemic and necrotic cells were seen in the cortex and cerebellum of groups receiving cells.

Excessive reactive astrocytes can produce pro-inflammatory and cytotoxic cytokines that are harmful to neurons or oligodendrocytes in the injured brain, and this may subsequently lead to more significant brain injury. An increase in GFAP concentration during astrogliosis has been demonstrated in several studies. GFAP is a sensitive marker for astrogliosis and increases immediately after traumatic brain injury (46). The immunostaining analysis indicated significant GFAP accumulation in all five different CP groups in comparison to the sham ones (Figure 3, *F* (4, 16) = 3.38, *P*=0.0345). This difference was more profound in the PND5, PND7, and PND14 groups (Figure 3). Also, to investigate the role of NPCs in astrocytes, we examined GFAP in the brains of CP rats. GFAP levels were considerably reduced in the groups that received the NPCs. Our findings indicate that the transplantation of NPCs led to a reduction in GFAP levels, which may play a role in regulating astrogenesis.

NeuN antibody was used to identify mature neurons in tissue sections, and it was considered to be a neuronal marker in the motor cortex. Two-way ANOVA showed no significant change in the NeuN marker among different groups (Figure 7, *P*=0.4009). Due to the hypoxic conditions in these three CP groups, specific neurons sustained damage, leading to a decrease in the overall number of neurons (Figures 7 and 8).

Neural progenitor cells can differentiate into neurons. In this experimental research, we investigated the differentiation of NPCs to mature neurons in the cortex of CP rats by analysis of the neuronal nuclear antigen (NeuN). NeuN immunohistochemistry is used in neuropathological studies to reveal their physiological status. In particular, immunoreactivity is significantly attenuated after severe injury, such as cerebral hypoxia/ischemia (47). Infants who have suffered from fetal stress or perinatal asphyxia may have lower NeuN immunostaining of the brain than infants who have not experienced such impairments (48). Our analysis shows an increase in NeuN level after transplantation of neural stem cells. This data confirms that the differentiation of NPCs to mature neurons is enhanced by NPC transplantation.

CD68 is another antibody used to identify a lysosomal protein commonly found in macrophages and activated microglia. In this study, we also used the CD68 marker to evaluate inflammation in the CP-injured samples. The significant presence of the CD68 marker indicates the activation of microglial cells and the occurrence of inflammation in the CP groups (48). 

Additionally, we analyzed the cluster of differentiation 68 (CD68) markers to confirm the decrease in inflammation following the transplantation of NPCs. Since CD68 is a recognized marker for activated microglia/macrophages, a decrease in this marker indicates reduced inflammation. Our result showed that CD68 was significantly lower in the group receiving NPCs, observed in the cortex and cerebellum of CP rats.

The cortex is one of the primary targets of CP pathophysiology. In the hippocampus of the CP group, degenerated neurons and apoptosis are detectable using imaging and pathological studies. Also, in the present study, H&E staining showed that the neurons in the hippocampus of the sham group were undamaged and intact. Previous reports have shown that following Ischemic hypoxia, the reduction of cerebral blood flow is a vital event that leads to structural, biochemical, and functional changes that ultimately cause irreversible death of neurons. Therefore, blood supply to the ischemic area is vital in preventing neuronal death (Figure 12).

This study examined neonatal rats at different ages (1–14 days old). As a result, some of the disabilities can be attributed to the lack of motor development due to the evolutionary process after the birth of neonatal rats. These disabilities were sometimes exacerbated in the sham groups, which can be mentioned as one of the limitations of this study. But in general, our study suggests that modeling CP similarly to humans can be a new strategy to help in understanding more about the mechanisms of CP in humans. As a result, it can be effective in developing therapeutic approaches. 

However, it is essential to note that interpreting these findings for human patients is still at an early stage. Animal studies provide valuable insights, but the complexity of the human brain and the challenges of clinical trials need to be considered. Further research needs to assess this approach’s long-term impact, safety, and efficacy in humans (49). However, the therapeutic use of NPCs in CP requires further research through clinical trials. Additional studies are needed to explore the long-term effects, optimal dosage, and potential side effects of stem cell therapy for brain disorders.

A section of the rat’s abdomen is shown on the left (a). A clamped uterine artery is shown on the right in cerebral palsy group (b). In the sham group, only the laparotomy was performed without uterine manipulation.

Scale bar = 50 µm.

**Table 1:** Hematoxylin & Eosin (H&E) staining of the hippocampus and ventricular area. On postnatal day (PND) 1, 3, 5, 7, and 14, the brain tissues of experimental neonatal rats were sectioned for histological evaluations which were grouped into five groups, sham groups (sham1-sham5) and cerebral palsy groups (cp1-cp5), respectively

*Groups*	*Sham-1*	*Sham-2*	*Sham-3*	*Sham-4*	*Sham-5*	*CP-1*	*CP-2*	*CP-3*	*CP-4*	*CP-5*
*Age (PND)*	P1	P3	P5	P7	P14	P1	P3	P5	P7	P14
*Anatomical Aria*	Hippocampus & Ventricle										
Pathologic Changes*	0	0	0	0	0	0	0	0	2	1
*Inflammation*	No	No	No	No	No	No	No	No	mild	No
*Necrosis*	No	No	No	No	No	mild	mild	mild	Moderate	mild
*Apoptosis*	No	No	No	No	No	No	mild	No	Moderate	Moderate

*0=without pathologic changes, 1=mild, 2=Moderate, 3=severe

N = five rats (5 rats for CP group and 5 rats for Sham group); Scale bar = 200 μm.

H&E: Hemotoxylin and Eosin, CP: Cerebral Palsy.

The brain tissues of experimental neonatal rats were sectioned for histological evaluations in CP and sham groups. There is an appearance of ischemia cells in the CP group, in contrast to a lack of ischemia cells in the sham group. In the details section, these changes are shown more clearly. N = five rats (5 rats for CP group and 5 rats for Sham group); Scale bar = 50 µm. CP: Cerebral Palsy, PND: Postnatal Day.

(a). On postnatal day (PND) 1, 3, 5, 7, and 14, the brain tissues of experimental neonatal rats were sectioned for histological evaluations in CP and sham groups. GFAP-positive cells significantly decreased in the CP-suspected groups. In the details section, the difference in GFAP expression levels between the two groups is shown more clearly. Data was statistically analyzed with two-way ANOVA followed by Sidak’s multiple comparison test, *P<0.05; **P<0.01; ***P<0.001; mean ±SED (b). N = five rats (5 rats for CP group and 5 rats for Sham group); Scale bar 100 μm. CP: Cerebral Palsy, PND: Postnatal day, GFAP: Glial fibrillary acidic protein.

**Table 2:** Pathological evaluations by Hematoxylin & Eosin (H&E) staining of sections prepared from the hippocampus and ventricular area in various PND. On postnatal day (PND) 1, 3, 5, 7, and 14, the brain tissues of experimental neonatal rats were sectioned for histological evaluations in cerebral palsy and sham groups. The scores are given based on the severity of the injury from lowest to highest (0-3)

**Groups**	**Sham**					**CP**				
**Age (PND)**	1	3	5	7	14	1	3	5	7	14
**Ischemia**	1	1	2	3	3	0	0	0	0	0
**Inflammation**	1	1	1	2	2	0	0	0	0	0
**Necrosis**	0	0	1	1	1	0	0	0	0	0

no evidence =0, severe=3

**Table 3:** Immunohistochemical staining of the rats’ Corpus Callosum (GFAP marker). On postnatal day (PND) 1, 3, 5, 7, and 14, the brain tissues of experimental neonatal rats were sectioned for histological evaluations in CP and sham groups (n=5)

**Groups**	**Sham**					**CP**				
**Age (PND)**	1	3	5	7	14	1	3	5	7	14
Proportion Score*	1	1	1	1	1	2	3	3	3	4
Intensity Score**	1	2	2	1	2	1	2	4	5	4
Allred Score (P+1)***	2	3	3	2	3	3	5	7	8	8

0= No cells are immunoreactive; 1= ≤1% of cells are immunireactive; 2= 1-10% cells are immunoreactive; 3= 11-30% of cells are immunireactive; 4= 31-60% of cells are immunireactive; 5= 61-100% of cells are immunireactive; **0= Negative; 1=Weak; 2= Intermediate; 3= Strong, ***0-2: negative; 3: Weak positive; 4-6: Intermediate positive; 7-8: High positive

**Table 4:** Immunohistochemical staining of the rats’ Cortex (NeuN marker). On postnatal day (PND) 1, 3, 5, 7, and 14, the brain tissues of experimental neonatal rats were sectioned for histological evaluations in CP and sham groups (n=5)

**Immunohistochemical staining of the Cortex** **(NeuN marker), n=5**										
**Groups**	Sham					CP				
**Age (PND)**	1	3	5	7	14	1	3	5	7	14
Proportion score*	5	5	5	5	5	4	4	4	4	4
Intensity score**	3	3	3	3	3	3	3	2	2	2
Allred score (P+1)***	8	8	8	8	8	7	7	6	6	6

*0= No cells are immunoreactive; 1= ≤1% of cells are immunireactive; 2= 1-10% cells are immunoreactive; 3= 11-30% of cells are immunireactive; 4= 31-60% of cells are immunireactive; 5= 61-100% of cells are immunireactive; **0= Negative; 1=Weak; 2= Intermediate; 3= Strong, ***0-2: negative; 3: Weak positive; 4-6: Intermediate positive; 7-8: High positive

(a). On postnatal day (PND) 1, 3, 5, 7, and 14, the brain tissues of experimental neonatal rats were sectioned for histological evaluations in CP and sham groups. In the details section, the difference in NeuN expression levels between the two groups is shown more clearly. NeuN positive cells percentages in Sham and CP groups at PND1, PND3, PND5, PND7 and PND14 (b). (F (4, 16) = 1.076, *P*=0.4009). Data was statistically analyzed with two-way ANOVA. N = five rats (5 rats for CP group and 5 rats for Sham group); Scale bar 100 μm. CP: Cerebral palsy, PND: Postnatal day.

Graphs show the NeuN markers decrease in the cerebral cortices of PND5, PND7, and PND14 CP-injured mice with respect to sham animals. (Unpaired t-test: PND5, t=3.560, df=8, *P*=0.0074; PND7, t=2.436, df=8, *P*=0.0408; PND14, t=3.345, df=8, *P*=0.0102). N = five rats (5 rats for CP group and 5 rats for Sham group). CP: Cerebral palsy, PND: Postnatal day.

(a). On postnatal day (PND) 1, 3, 5, 7, and 14, the brain tissues of experimental neonatal rats were sectioned for histological evaluations in CP and sham groups. CD68-positive cells were detected in high numbers in the CP groups, while markers were rarely found in the sham groups. In the details section, the difference in CD68 expression levels between the two groups is shown more clearly. Statistically analysis with two-way ANOVA followed by Sidak’s multiple comparison test, *P<0.05; **P<0.01; ***P<0.001; mean ±SED (b). N = five rats (5 rats for CP group and 5 rats for Sham group); Scale bar 50 μm. CP: Cerebral palsy, PND: Postnatal day.

**Table 5:** Immunohistochemical staining of the rats’ Corpus Callosum (CD68 marker). On postnatal day (PND) 1, 3, 5, 7, and 14, the brain tissues of experimental neonatal rats were sectioned for histological evaluations in CP and sham groups (n=5)

**Groups**	**Sham**					**CP**				
**Age (PND)**	1	3	5	7	14	1	3	5	7	14
Proportion score*	2	2	2	2	2	3	3	3	3	3
Intensity score**	2	2	3	3	2	2	3	3	4	4
Allred score (P+1)***	4	4	5	5	4	5	6	6	7	7

*0= No cells are immunoreactive; 1= ≤1% of cells are immunireactive; 2= 1-10% cells are immunoreactive; 3= 11-30% of cells are immunireactive; 4= 31-60% of cells are immunireactive; 5= 61-100% of cells are immunireactive; **0= Negative; 1=Weak; 2= Intermediate; 3= Strong; ***0-2: negative; 3: Weak Positive; 4-6: Intermediate Positive; 7-8: High Positive

(A, B). Scale bar: 100 μm. NPCs : Neural Progenitor cells

**Table 6:** Immunohistochemical staining of rats’ cortex and corpus callosum area in CP and Sham groups. On postnatal day (PND) 1, 3, 5, 7, and 14, the brain tissues of experimental neonatal rats were sectioned for histological evaluations which were grouped into five groups, sham groups (sham1-sham5) and cerebral palsy groups (cp1-cp5) , respectively

*** Groups***			**Sham-1**	**Sham-2**	**Sham-3**	**Sham-4**	**Sham-5**	**CP-1**	**CP-2**	**CP-3**				**CP-4**			**CP-5**
* Age (Day)*			P1	P3	P5	P7	P14	P1	P3		P5	P7			P14		
*Staining*	GFAP	Anatomical aria	Cortex														
		Proportion score*	1	1	1	3	3	2	3		3		3			4	
		Intensity score**	1	2	2	1	2	1	2		4		5			4	
		Allred score (P+1)***	2	2	3	4	5	3	5		7		8			8	
		Microgliosis	No	No	No	No	No	Slight	Slight To Moderate		Slight To Moderate		Moderate			Moderate To High	
	Neun	Anatomical aria	Cortex														
		Proportion score*	5	5	5	5	5	5	5		5		5			5	
		Intensity score**	3	3	3	3	3	3	3		3		3			3	
		Allred score (P+1)***	8	8	8	8	8	8	8		8		8			8	
	CD68	Anatomical aria	Cortex														
		Proportion score*	2	2	2	2	2	3	3		3		2			3	
		Intensity score**	3	3	3	3	2	3	3		3		2			3	

*0= No cells are immunoreactive; 1= ≤1% of cells are immunireactive; 2= 1-10% cells are immunoreactive; 3= 11-33% of cells are immunireactive; 4= 34-66% of cells are immunireactive; 5= 67-100% of cells are immunireactive; **0= Negative; 1=Weak; 2= Intermediate; 3= Strong; ***0-2: negative; 3: Weak positive; 4-6: Intermediate positive; 7-8: High positive

**Table 7:** Pathological evaluations by Hematoxylin & Eosin (H&E) staining of sections prepared from cortex area in received cells group and without injection (sham) group. The scores are given based on the severity of the injury from lowest to highest (0-3)

Hematoxylin & Eosin (H&E) staining (cortex)			
Groups	Received cells group	Without injection (sham)
Ischemia	0	2
Inflammation	1	3
Necrosis	0	1

No evidence =0, severe=3

Ependymal lining was thickened, indicating injury in the group no receiving cells. There were less neuronal damage and inflammation in the group receiving cells compared to another group. The Scale bar is 50 μm. H&E: Hemotoxylin and Eosin, CP: Cerebral palsy, NPCs :Neural progenitor cells.

(a). GFAP and CD68 expression were lower in the group that received cells compared to the CP group. NeuN expression was lower in the CP rats compared to the transplanted groups. CP rat injected with the NPC (left column) and CP rat without injection (right column). Statistically analysis with two-way ANOVA followed by Sidak’s multiple comparison test (b), *P<0.05; **P<0.01; ***P<0.001; ****P<0.0001; mean ±SED. N= five rats (5 rats for CP group and 5 rats for Sham group); Scale bar 50 μm. CP: Cerebral palsy, NPCs: Neural progenitor cells, GFAP: Glial fibrillary acidic protein.

## Conclusion

In general, the results of this study indicated that using hypoxia by blocking the uterine artery can create a model of CP in neonatal rats. Therefore, this approach can be a relatively low-cost and straightforward method with the desired efficiency in future studies. Also, our results showed that neural progenitor cells promise to replace damaged brain tissue in CP, improve symptoms, and enhance function. This could be a promising treatment option for CP patients.

## Data Availability

The raw data supporting the conclusions of this article are available from the corresponding author upon reasonable request.

## Declaration

There is no financial affiliation between any of the writers and any business that has an interest in the topic of this work. We acknowledge the use of Wordadvice AI to enhance the language and readability of the manuscript. Other aspects, such as data analysis, the production of images and graphs, and so on, were carried out without the intervention of artificial intelligence.
